# The Importance of Religiosity/Spirituality in the Sexuality of Pregnant and Postpartum Women

**DOI:** 10.1371/journal.pone.0156809

**Published:** 2016-06-16

**Authors:** Sagrario Gómez Cantarino, José Manuel de Matos Pinto, Joana Alice da Silva Amaro de Oliveira Fabião, Ana Maria Carrobles García, Minerva Velasco Abellán, Manuel Alves Rodrigues

**Affiliations:** 1 Departamento de Enfermería, Fisioterapia y Terapia Ocupacional, Escuela de Enfermería y Fisioterapia, Campus Toledo, Universidad de Castilla la Mancha, Toledo, España; 2 Health Sciences Research Unit: Nursing, Nursing School of Coimbra, Coimbra, Portugal; 3 Departamento de Enfermería, Fisioterapia y Terapia Ocupacional, Escuela de Enfermería y Fisioterapia, Campus Talavera de la reina, Universidad de Castilla la Mancha, Toledo España; Catholic University of Sacred Heart of Rome, ITALY

## Abstract

In this article, we decided to study the representation of the Spanish pregnant and postpartum women and the importance of religiosity/spirituality and the social context for them. We analyzed the influence of religion on the woman within her social context. **Objective**: to understand how pregnant and postpartum women experience their sexuality according to their religious beliefs and the opinion of others from a socially learned perspective. **Method**: qualitative study using ethnography. This study aims at understanding reality from the women’s point of view, acknowledging that the points of view are constructed through interaction with others, through cultural and historical norms that influence the lives of individuals. **Results**: The findings indicate a difference in the religious beliefs and sexual behaviors of these women, which is more marked in urban than rural areas. Mothers have an influence on their daughters, conditioning their behavior. **Conclusion** We conclude that the process of change is underway. However, some paradoxes still persist concerning the sexual roles to be adopted, as well as some contradictions between sexual behaviors and the statements on religion. Within the scope of our study, we can confirm that pregnant and postpartum women are more or less pressured by the religious and social norms conveyed by their mothers, mainly in rural settings. From an external point of view, to be sexually free goes against the maternal and social expectations. However, the internal representation, marked by religion, that has been experienced over the years does not change the narratives of sexual experiences, assigning women to traditional role. This role brings conflict more or less assumed by women.

## Methodology

This study was approved by the Clinical Research Ethics Committee of the Hospital of Toledo, belonging to the Health Service of Castilla-La Mancha. This Committee also approved the informed consent form that should be signed by all participants. Both the study and the informed consent form were approved on June 24th, 2009. (CEIC SALIDA n° 49).

This is a qualitative and exploratory study with an ethnographic research design developed within the constructivist paradigm. This design was used because it focuses on understanding and further exploring the importance of the sexual experiences of a group of rural/urban pregnant and postpartum women and their mothers according to their religious/spiritual beliefs. Based on the traditionally realistic ethnographic perspective [1], we will objectively describe what we observed and what the participants reported, trying not to manipulate or modify the natural scenarios [2], valuing the meaning assigned by these women to their own actions, which reflect the cultural path of socialization. For this reason, the data obtained and the opinions of the participants are shown in quotations, as accurate as possible.

According to the constructivist paradigm, there are multiple realities which are built at an individual and social level in such a way that social life unfolds based on the social and cultural norms in a given context and at a given moment in time [3].

The purpose of this study is to understand the importance of religiosity/spirituality in the sexuality of pregnant and postpartum women in several situations of their social development. Here, the health care centers are considered to be ideal spaces for getting together and sharing experiences.

### Sample selection

The following inclusion criteria were used: a) being over 18 years of age, b) being in the second or third semester of pregnancy, c) being in the postpartum period or breastfeeding, d) having received prenatal care, maternal education and advice on breastfeeding in the health care centers participating in this study, and e) being fluent in spoken Spanish language.

The following exclusion criteria were used: a) women in the first trimester of pregnancy, b) having a pre-pregnancy pathology, and c) women with communication difficulties.

Intentional sampling was used to select and recruit the participants, allowing for the selection of the women and health care centers that were able to provide more and better information. Data saturation was achieved when the additional interview to the women brought no new insights to the information already obtained. [2]. It is important to underline that these type of studies use small samples, whereas the qualitative methods often produce results that can be shared by a community or social group [4].

After asking the women to fill out a personal information sheet, the researchers established the first telephone contact with each of them to schedule the date and time for the interview.

### Informants

**Fig 1:**
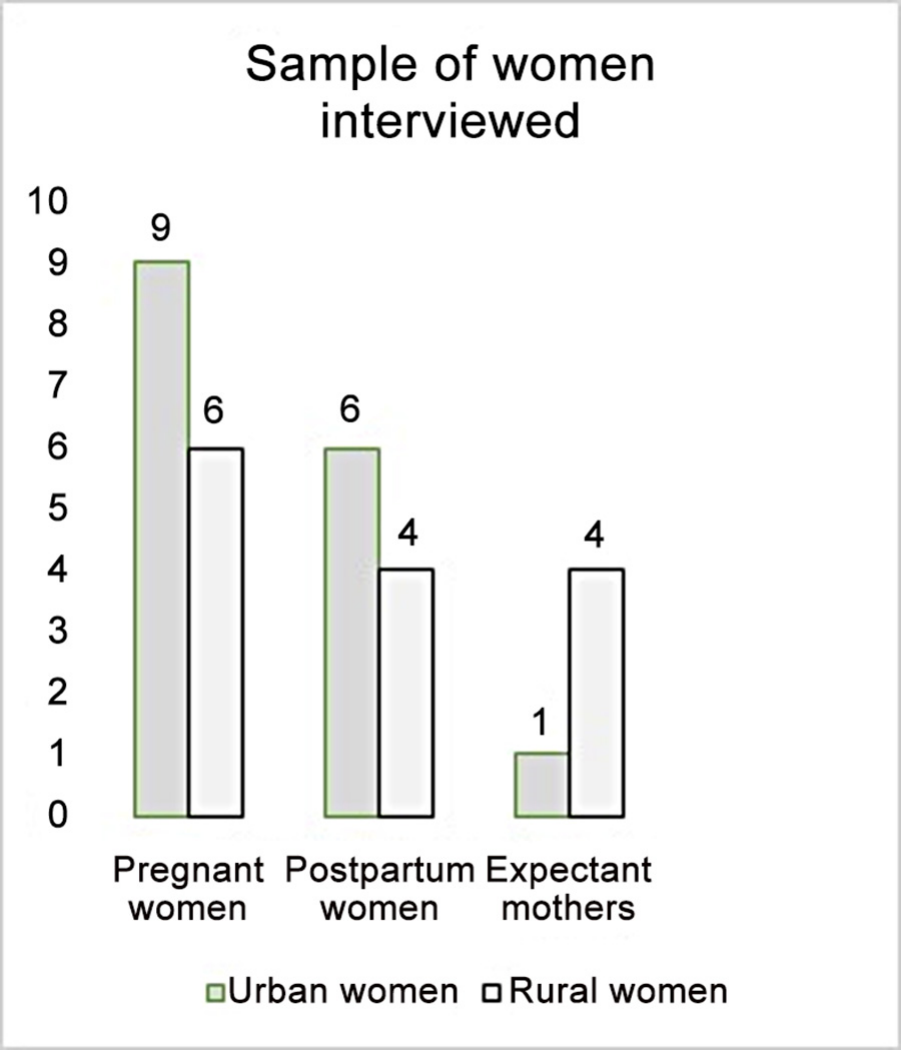
Sample of women interviewed. Source: Created by the authors.

The participants were users of two health care centers located in an urban and in a rural area: pregnant women (15), postpartum women (10), mothers of pregnant women (5). These health care centers belonged to the health area of Toledo, the Health Service of Castilla La Mancha (SESCAM). (Fig 1).

### Scenario

The study was conducted in two health care centers (urban and rural) of the Health Service of Castilla-La Mancha (SESCAM), in the province of Toledo, in the No. 1 Health Area.

According to the Regional Government of Castilla La Mancha (Spain), in 2010, there are 14 274 inhabitants in this core area, of whom 7126 are women. In this area, there are 4 328 women of child- bearing age (15–49 years) and 1 122 pregnant women.

The urban health care center (Buenavista), with autonomous management, treated 10 565 women, of whom 8 942 of them were women of reproductive age [5].

In the rural health care center (Villaluenga), most women are aged between 20 and 49 years (≃ 910). [5]

### Data collection techniques used

The most important techniques in the ethnographic method were used: participant observation and in-depth interview (Table 1).

**Table 1:** Collection of technical information.

Interviews	Observation (Venue)	Other Sources Written Information)
Pregnant women	Urban and rural Health Care Center	Official documents, informative documents, newspaper articles, radio show
Postpartum women	Urban and rural Health Care Center	Official documents, informative documents, newspaper articles, radio show
Mothers	Urban and rural Health Care Center	Official documents, informative documents, newspaper articles, radio show

Source: Created by the authors

The in-depth interview was used because through questionnaires or closed instruments we could not further explore the topic under analysis in this exploratory study. This method puts women in a dynamic and recognized position. A series of questions were posed in which their composition or order was not intended to be strict and immutable [6]. In-person meetings were held between the main researcher and the interviewed women with a view to understanding the women’s opinions about their sexual lives and experiences from a religious/spiritual perspective. The principal investigator who conducted the interviews was an experienced researcher given her care practice and approach towards the women. The interviews took place in the health care centers where the study was conducted, which may have facilitated the meetings between the researchers and the women to be interviewed. The interviews were open, dynamic, flexible and prolonged, using a script that reoriented the interview for obtaining relevant information for the study. The interviews lasted between approximately 45 minutes and an hour and a half. Women were informed that the notes made during the interview would not identify them in any case.

All women received information from the principal investigator and signed the informed consent before participating in the study.

In the health care centers, participant observation was conducted, in: the sessions attended by women; the waiting rooms; and even the immediate surroundings, through the conversations between the women and their mothers. We tried to develop the daily activities in the most natural way possible [7]. The purpose was to identify the religious/spirituals beliefs and the involvement of these women in their sexuality, trying to assess their opinions. We did this with some flexibility, i.e. based on the previous categories for observation (ETIC) and incorporating those from the women, since those from the researchers were not enough to explain the events (EMIC). Observation was performed for some time and on different days of the week to compare the narratives with the practices in different situations. We visited each place in more than one occasion. A total of 52 visits were performed to the venues of the ethnographic area, in a total of 208 hours of observations.

The in-depth interview and the participant observation are techniques that complement and reinforce each other, making it possible to obtain accurate and reliable data.

Since this qualitative study with an ethnographic focus was conducted in natural scenarios, if another researcher performed the same study, he/she would probably obtain different data due to the dynamic condition of human behaviors.

An official letter describing the study was initially sent to the Management and Executive Boards of the health care centers where it was developed.

We started conducting the study on the field on June 10, 2009, ending this process in December 2010.

### Analysis

This global, holistic, complex and changing study with a flexible and emergent design should lead to accurate and reliable data.

**Fig 2:**
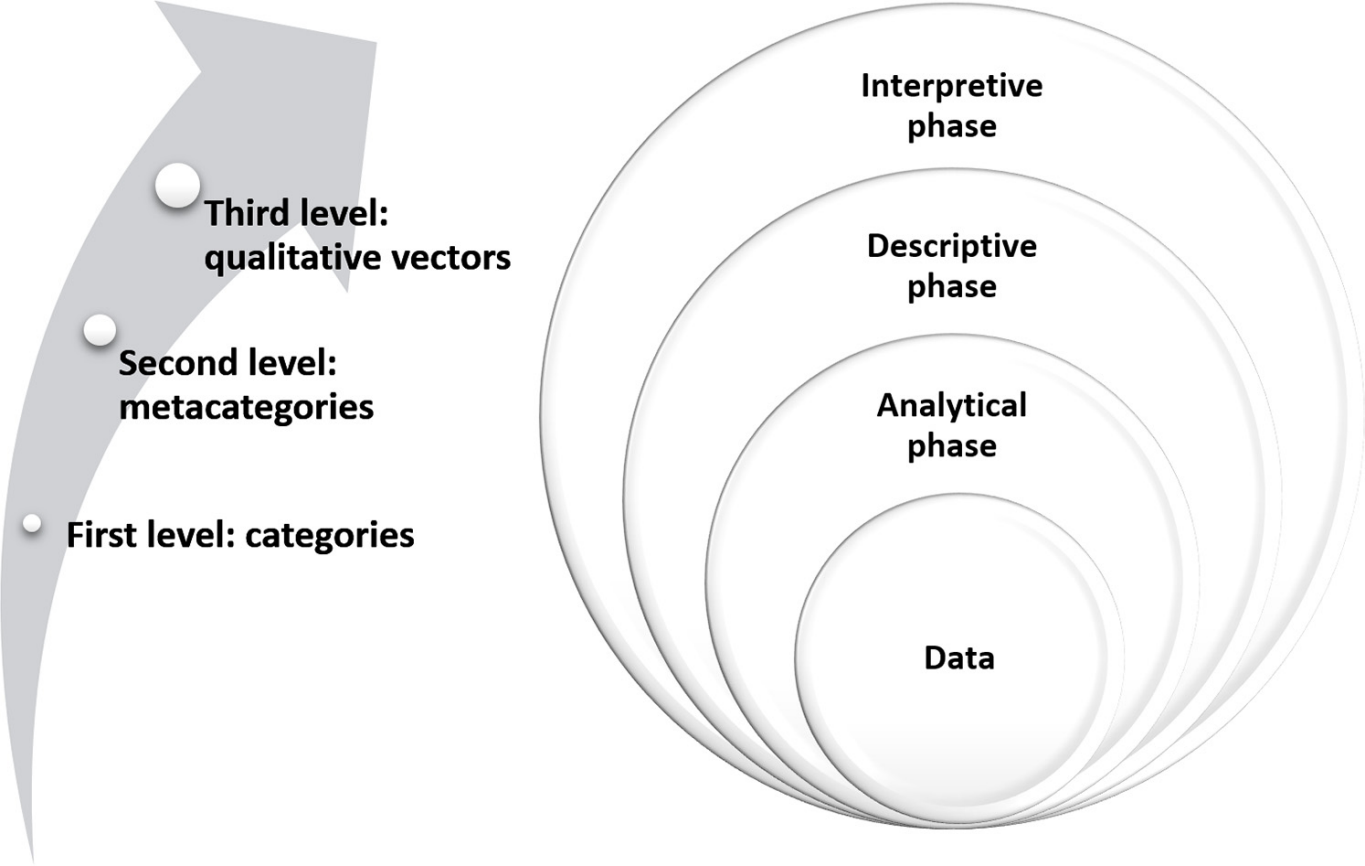
Analytical phase. Open and Axial Coding. Source: Created by the authors.

To this end, we used the constant comparative method, which served as a guide for the development of the analytical process. This method is not exclusive to a single type of research tradition, being able to be applied in ethnographic studies similar to this [8]. Therefore, three levels of analysis were established: open coding, axial coding through constant comparisons, and selective coding [9] (Fig 2).

Open coding, as the first level of analysis, consisted of dividing the information collected from the interviews, the observation and the analyzed documents into smaller significant parts and comparing and grouping similar categories. The purpose was to find relevant units or cores of meaning. For this, we used the Atlas. ti (6.0) software, since it facilitated the systematization of data sorting and processing [9], and allowed grouping the units for coding.

Axial coding, as the second level of analysis, tries to identify associations between categories and regroup them into meta-categories [10], performing this in more depth after ending the fieldwork.

**Fig 3:**
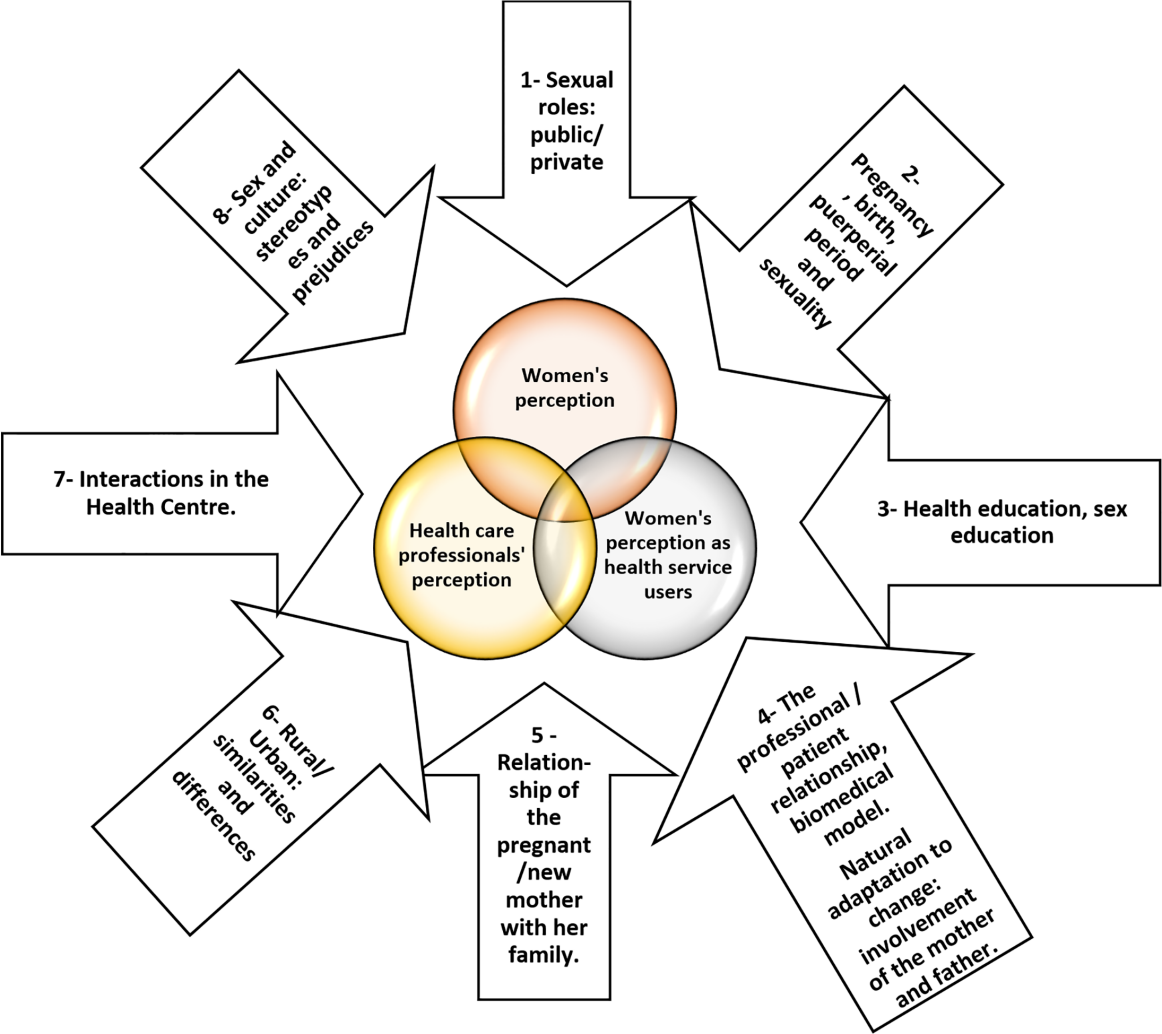
Graphic representation of meta-categories/qualitative vectors. Source: Created by the authors.

Selective coding, as the third level of analysis, corresponds to the identification of qualitative vectors. In this study, three qualitative vectors were identified [9] (Fig 3).

In this study, we applied the following quality criteria listed below [11–12].

Reliability: so that the interviews’ transcripts were complete and thorough. We distinguished between the emic (our informants) and the etic (the researchers) perspectives.

Possibility of confirmation: the transcripts of each interview were validated by the interviewee, allowing women and their mothers to decide if the issues addressed were correct and any aspects to be clarified.

Meaning in context: based on the informants’ narratives from their own perspective (emic), these informants describe their situation and the world of their experiences.

Patterns of meaning: this brings us to the units of meaning that emerge in the interviews (emerging categories). Our starting point was a guide of categories.

Saturation: It was achieved when the data added nothing new and the ideas were duplicated.

Possibility of transfer: It is complicated from the point of view of replicability, since the study was conducted in natural scenarios. It is suggested that the exact duplication of the methods cannot produce identical results [8–9–11].

## Introduction

This is an ethnographic study with a sample of Spanish pregnant and postpartum women, focusing on the association between sexuality and religion/spirituality. The purpose was to understand the influence of religion on pregnant and postpartum women within their social context.

The interest of this study lies in describing the sexual experiences of pregnant and postpartum women and their association with religious/spiritual beliefs, from a socially learned perspective. Spirituality has been historically associated with religion. Religion is an element of spirituality. Therefore, we can state that religiosity and spirituality have had a fruitful and contradictory role, because, although most health professionals show a low level of interest for religious matters, the populations have beliefs, practices and experiences related to religion [13]. For this reason, it is important to understand these concepts. The word religion derives from the Latin word *religio*, which means union (reconnect) between humanity and transcendence (more human) [13]. Thus religion allows individuals to attach a meaning to their beliefs, experiences and practices in different life situations. In this case, religion would be a sacred source of motivation, sense and meaning, i.e. the capacity to build resistance and cope with the adversities that can influence the health of individuals [14–15] and their behavior [16]. Positive psychology is currently discussing “the positive role of religiosity/spirituality in people’s lives and in the social organization (…) [stating] that it contributes to the development of positive, healthy aspects, such as hope, faith, self-esteem and optimism, among others” [17]. On the other hand, religiosity/spirituality seems to be related to more protective and less risky sexual behaviors, which is an important aspect for pregnant women when they are not married or in the expected cycle of maturation and reproduction [16–18–17]. This leads to an increased focus on reading oneself and one's social and sexual behavior.

Sexuality is a complex process which may lead to increased well-being and satisfaction within the relationship [19–20–21–22–23], but which may involve risks of self-reported dysfunction [22].

Although we find accounts of women associating sexuality with higher levels of satisfaction, some accounts also report difficulties associated with sexuality, such as those related to the very restrictive sexual norms imposed by religiosity/spirituality [24]. Similarly, we can say that sexuality seems simple only in appearance, and as an example, we present a detailed analysis of major difficulties and dysfunctions in men and women. A study with a sample of 43 male adolescents and 128 female adolescents (aged 17–21 years) and 28 male young adults and 65 female young adults (aged 22–28 years) found few differences in the sexual difficulties reported in terms of interest, arousal, orgasm, pleasure, and fear. The answers indicated high levels of desire, pleasure and satisfaction, and also life experiences with sexual difficulties and problems. [25].

On the other hand, there are reports of a significant loss of sexual pleasure during pregnancy. It is thus important to understand how pregnant and postpartum women experience their religious beliefs and the opinion of others about themselves from a socially learned perspective.

## Results and Discussion

### 1. Prejudice

Based on our observations throughout the study, we found that the women are reluctant to address sexual issues, which is related to a situation of prejudice influenced by thoughts of “what would they say or think” deeply rooted in the religious concepts existing in the study area. There seems to be a code of conduct to which they must adhere to, which is manifested within the cultural and historical context that influences these women’s lives. One of the pregnant women in the rural area said “I rarely talk about this, we don’t address it, it has been this way forever” [Field note, July 8, 2009]. Another pregnant woman during a workshop in the same rural area said: “these things, we don’t say them or talk about them, we have been doing them but hiding them, out of respect, it is normal” [Field note, September 3, 2009] (Table 2).

**Table 2:** Socio-demographic characteristics of the sample: urban and rural pregnant women.

Variables	Urban and rural pregnant women
**Age**	
20–25	3
25–30	3
30–35	9
**Marital status**	
Married	12
Cohabiting	3
**Educational level**	
Secondary education	4
Higher education	5
Technical career	6
**Occupation**	
Homemaker	3
Domestic worker	2
Retail employee	4
Business employee	6
**Gestational Age**	
28–31	4
32–35	8
36–39	3
**No. of pregnancies**	
1	5
2	8
3 or more	2
**No. of children**	
1	8
2	5
3	2

*Note*: N = 15

Source: Created by the authors

It should be noted that the Spanish society from the 1950s to the 1980s was strongly influenced by the religious issues, with this still being a reality today. This is even more consolidated in the mothers of rural pregnant women. [26–27]. These mothers have been strongly conditioned by the existing social morale at the time, which has been passed on to their daughters, who have sometimes hidden, as pregnant women, relationships, feelings, and actions due to the existing prejudice in the area where they live. Their society has guided them to correct or good sexual behaviors and, in the same way, to incorrect or poor behaviors.

Accounts collected during an interview from one of the mothers:

[MGR12 Sofia, rural pregnant mother]: "…they told us that feeling pleasure…while we were pregnant…that was not right…that was vulgar and especially shameful…and so we hold back…”

In some occasions, these women hide relationships, feelings and actions due to the existing prejudice where they live. Some of the women in this study even reported that maintaining sexual relationships with their partner at this time was impossible.

[GU12 Sofia, rural pregnant woman] "…we don’t agree with it, he more than me, he thinks that’s not right, the child inside and we…no, even thinking about it, that's not right…"

[GU11 María, rural pregnant woman]: "…in my case, I have no need of a man now that I'm pregnant…look, to avoid the worst, I agree when he cannot hold it any longer, and so he remains calm and satisfied…but no more…we shouldn’t be doing this in my situation…"

Here, we can see how religion and spiritual activities may promote prejudice. The fact of women being able to express their sexuality is criticized and ill-considered without having enough information to substantiate the critique. [28]. This situation is a reality in both rural and urban areas of this study. In an urban workshop on breastfeeding, one of the postpartum women said “it may seem that we are freer for having sex, but that is not right, there are rules to follow”. [Field note, March 16, 2010]. Here we can see the existence of that type of situations such as love, feelings and including pregnancies because they are not well considered in their social context.

In the following account on sexuality and pregnancy, it is made clear that pleasure is totally inappropriate during this period: "Women are not supposed to have sexual needs during pregnancy. If they had them, they should be imperatively rejected for the benefit of the development of maternal love whose nobility cannot suffer an immoral competition.” [27].

### 2. Religion and society

It should be noted that there the catholic ideology in the study area until the 1990s had reinforced the ideal of the pure and, above all, formal woman. Therefore, in the studied sample, religion is consistent with the socialization of the women, with marriage being the ultimate goal.

A postpartum woman reflected on the topic of sexual relationships with the partner during an interview (Table 3):

[PP9 Rosa, rural puerperal woman]: "…I have to do it, I am his wife and I'm ready to restart the sexual relationships with my partner…I still feel uncomfortable, but he is already asking me…and I need to know which birth control method can I use…”

**Table 3:** Socio-demographic characteristics of the sample: urban and rural postpartum women.

Variables	Urban and rural postpartum women
**Age**	
20–25	4
20–25	4
25–30	1
30–35	1
**Marital status:**	
Married	9
Cohabiting	1
**Educational level:**	
Secondary education	2
Higher education	6
Technical career	2
**Occupation**	
Homemaker	2
Domestic worker	1
Retail employee	1
Business employee	6
**N°o. of pregnancies**	
1	6
2	3
3 or more	1
**No. of children**	
1	5
2	4
3	1

Source: Created by the authors

*Note*: N = 10

The church believed that these were not two different people but a single individual, so the interests, aspirations, friends and preferences should be the same, which is required by sharing a life together. [29] Thus "religion would play an irrational role in modern society: the salvation from individual suffering and sin. (…) [In such a way that it would not be an installed conflict] for the church, it is procreation, but nevertheless the erotic sphere values pleasure". [24].

It should be noted that the issue of sexuality addressed in the workshops is extrapolated to the exterior without complexes, leading the women to find a way out, ask for help, even trying to be honest with themselves. One of the postpartum women said: “if you have recovered, and your body asks it, and you’ve been waiting for it a long time, try it and lay down with him because he’s your husband” [Field note, September 17, 2009]. In rural areas, where there isa more socially restrictive control, the rejection of sexual pleasure in marriage is more internalized than in urban areas, but not inexistent. [30]. In these women, it is very important to know the birth control method that they should be using to prevent another pregnancy, which made some of the women in this study develop contradictory feelings, since their behaviors challenged their Christian moralist convictions. A midwife of the rural area said: "…now you have to take care of you and use the condom that is safe, effective and comfortable…". [Field note, September 15, 2009].

In relation to the pain felt at the beginning of sexual intercourse with penetration, we found that these women have reasons of complacency towards their partner, as can be seen in this interview excerpt:

[PP7 Rocio, rural postpartum woman]: "…I think that I won’t be able to…my sister told me that it was very hard for her…until the third time she couldn’t…, oh my God, I have tried it once…and I wasn’t able…when my husband ask me to try again…we’ll see, but my body is not ready…"

Regarding the discomfort in the initiation of sexual intercourse among the women in this study, the midwife of the rural area, who is involved in education and counseling, and respecting the social values, answered "…well…look, vaginal lubricants help…that is until penetration…then…slowly…". [Field note, April 20, 2010].

According to Durkheim’s social perspective, which regards religion as a powerful moral force that induces conformity, some authors believe that religion would act as an agent of socialization, by allowing social interaction and attachment through the religious practice of the cult [31–32].

On the other hand, it is clear that the issues on sexuality were currently being addressed by the health professionals working at the health care centers where the study was developed. The topic is widely accepted by the pregnant women and even by their accompanying mothers, possibly because it provides a more inclusive and less moralist perspective than the religious approach to sexuality.

It is still inferred that religion attaches great weight to sexuality in the beliefs and social identities. Faith and religious practice are associated with a more conservative sexuality and, consequently, a less risky sexual behavior. So, in our study, the traditional family is preserved in the rural area, as can be seen in the following interview:

[GU2 María Jesus, urban pregnant woman]: "…I still had a year of studies left, but my boyfriend was leaving…I went with him, I didn’t think about it, I was not going to lose him…but before I had to go the vicarage".

We found that the subject of marriage, traditionally deeply rooted in this study area, becomes a secondary issue, seen in this way by younger women, because there is among them a tendency to social change, as they refuse to comply with the moral and traditional norms surrounding them: it seems that the meaning assigned to the sexual experience is changing, although some obvious or underlying conflicts with previous behavior models still exist. These conflicts can be seen in the emerging sexual difficulties and are reported by these young women, which is in line with the difficulties reported by several authors. [22–23]. An example of this situation is the following interview excerpt:

[GR13 Jenifer, rural pregnant woman]: "…when I said that my idea was to go live with him, my parents almost fainted, they are very traditional people, and…to think that their daughter lives in sin…or about what people will say about her…they couldn’t bear it…".

We perceived a process of change in the beliefs and social behaviors and, consequently, in sexual behaviors, which is more marked in urban than in rural areas, indicating a liberalization and a more complete experience of sexuality among Spanish young women.

### 3. Past, present and future. A new perspective

The mothers of the women participating in this study were attracted by it and saw themselves immersed in it. T they have openly expressed their valuable opinion, since they were the ones educating their daughters. The following accounts help us understand their interventions, opinions and experience (Table 4):

[MGR14 Andrea, mother of a rural pregnant woman]: "…it is because we knew nothing, and when you got married you were so young…that whatever happened was ok…I didn’t care if I had sexual desire, if he had it…all was said, I had to and…then, I liked it…"

[MGR11 Paca, mother of a rural pregnant woman]: "…the problem was that there was no way of not getting pregnant, and withdrawal…It left you sometimes very sick…and if there were any methods, we didn’t get them…and you had to endure, as it was also wrong to use them…it was a sin…that is, it was frowned upon…”

[MGR11 Paca, mother of a rural pregnant woman]: "…and if you refused it didn’t matter…you knew that you had to do it and that’s it…it did not matter if you didn’t want it…”

**Table 4:** Socio-demographic characteristics of the sample: mothers of urban and rural pregnant women.

Variables	Mothers of urban and rural pregnant women
**Age**	
50–55	1
55–50	3
60–65	1
**Marital status:**	
Married	4
Widowed	1
**Educational level:**	
Elementary	3
Secondary	2
Technical Career	0
**Occupation**	
Homemaker	4
Domestic helper	1
**No. of children**	
1	1
2	1
3 or more	3

Source: Created by the authors

*Note*: N = 5

These statements seem to reinforce the women’s subordination to a reproductive role in their marriage and family.

The descriptions given by the women in our study seem to characterize men as being more impulsive and sexually active. A woman in the breastfeeding workshops stated that “my man doesn’t wait right after the 3 a.m. breastfeeding, he wants sex; let’s start, he doesn’t care, so I have to” [Field note, February 3, 2010]. Several authors mention that there are several factors influencing sexual desire, including gender. Women seem to have less sexual desire than men, due to a lower biological need for sexual approach [33–34] or because they have learned to repress their sexuality [35–36].

As previously mentioned, men are more eager and impulsive in their sexual life as women, who are more passive. Here is another comment obtained during the breastfeeding workshops: “Now I have other obligations, I don’t think about having sex so often, I don’t have time, but he hasn’t change and he asks me to have sex” [Field note, March 16, 2010]. Outercourse is poorly tolerated by the partner, as men need to have sex frequently, while women, in general, only accept to have sexual intercourse because their partners, not them, have expressed this need. A pregnant woman comment on this in the workshop: “He doesn’t want just the for play, we need to have sex, but not for me [Field note, March 9, 2010].

These women also reported feeling more relaxed when having sexual intercourse while pregnant, as in the account “I’m on my third pregnancy and how I do it but in a more relaxed way” [Field note, March 23, 2010]. It can be seen here that it was a time when they obviously could not get pregnant again. Some of the women in our study used breastfeeding as a possible birth control method, complying with their spiritual commitment and the rules of their religion. This issue is addressed in the following interview excerpt:

[Pp3 Isabel, rural postpartum woman]: "…well, I'm not using any birth control method…but I’m concerned…I'm breastfeeding and menstruating…every month! And of course I think it's difficult to get pregnant in this situation…but the important is that I breastfeed…"

Hence, we confirm the importance of socialization in this group of women, since they repeat the practices performed by their mothers, such as using breastfeeding to avoid getting pregnant. To illustrate this, we present another account collected during an interview from one of the mothers:

[Ppr4 Ana, rural puerperal woman]: "…I knew nothing; I always thought that I was protected while breastfeeding, that's what they told me, even my sister-in-law told me that!"

In short, we can say that the perspective of sexuality in these women includes sexuality, pregnancy and breastfeeding, as they even perceive breastfeeding as a “birth control method”. This conservative perspective perpetuates the male and female roles that were conveyed by their own mothers, as well as preserve their religious beliefs.

## Conclusion

We conclude with this study that the process of change is underway, although some paradoxes persist concerning the sex roles to be adopted, as well as some contradictions between religious beliefs and sexual behaviors. Within the scope of our study, we can say that pregnant and postpartum women are pressured by the religious or social norms verbalized by their mothers, mainly in the rural context where discourse does not always match the practice, imposing on pregnant and postpartum women a set of beliefs that change their sexual experience

In short, there is still much to change and integrate regarding the sexual roles of pregnant and postpartum women. The health care professionals play a very important role in demystifying some restrictive sexual beliefs for religious reasons, which may cause some discomfort among pregnant and postpartum women.
